# An Observational Cross-Sectional Study on the Correlation between Professional Competencies and Self-Efficacy in Albanian Registered Nurses

**DOI:** 10.3390/healthcare11152156

**Published:** 2023-07-28

**Authors:** Blerina Duka, Alessandro Stievano, Emanuela Prendi, Florian Spada, Gennaro Rocco, Ippolito Notarnicola

**Affiliations:** 1 Department of Biomedicine and Prevention, University of Rome “Tor Vergata”, 00133 Rome, Italy; bleriduka@yahoo.it (B.D.); e.prendi@unizkm.al (E.P.); f.spada@unizkm.al (F.S.); 2 Faculty of Medicine, Catholic University “Our Lady of the Good Counsel”, 1000 Tirane, Albania; alessandro.stievano@gmail.com (A.S.); genna.rocco@gmail.com (G.R.); 3 Centre of Excellence for Nursing Scholarship, Order of Nursing Professions (OPI), 00173 Rome, Italy; 4 Department of Clinical and Experimental Medicine, University of Messina, 98122 Messina, Italy

**Keywords:** cross-sectional study, observational study, nurse, nursing, professional competence, self-efficacy

## Abstract

(1) Background: The assessment and application of registered nurses’ professional skills are essential to providing quality and safe care. Self-efficacy can positively affect the professional competence of registered nurses. This study analysed professional competence and its association with self-efficacy among registered nurses. (2) Methods: A cross-sectional observational study was conducted. The sampling was conventional. The data collection took place through the Albanian version of the Nurse Professional Competence Scale Short Form (A-NPCS-SF), which was used to assess their professional skills, and the Albanian version of the Nursing Profession Self-Efficacy Scale (A-NPSES), which was used to assess their self-efficacy. The study was based on a convenience sample of 985 registered nurses from the 12 Albanian provinces. (3) Results: The Cronbach alpha value for the A-NPCS-SF scale was 0.947, while for the A-NPSES scale, it was 0.875, proving both scales to be reliable. Self-efficacy does not play an essential role in the development of the professional competence of registered nurses since our survey found only one dimension correlates with these two elements. (4) Conclusions: The results of our analysis have instead highlighted the importance of a close relationship between job satisfaction and the development of professional skills.

## 1. Introduction

Self-assessment helps nurses maintain and improve their practice by identifying their strengths and areas that need further development. Professional competence profiles encourage them to participate in continuing education and active learning. Although the recognition of competence provides a way to motivate nurse practitioners to produce quality care, few measurement tools are available for this purpose [1]. In social cognitive theory, self-efficacy is described as an individual’s belief in their ability to succeed at a specific task or accomplish a specific goal [2].

In other words, self-efficacy is the belief in one’s abilities to organize and execute the actions necessary to manage challenging situations. Self-efficacy is a psychological construct closely related to self-esteem, optimism, and self-mastery; it differs from other theoretical concepts because it is explicitly concerned with people’s belief in their ability to achieve goals [3]. During the last two decades, the literature has highlighted several reasons why the assessment and promotion of self-efficacy are essential for some professional groups [4,5,6,7,8,9].

High self-efficacy is primarily associated with a greater sense of control, motivation, and resilience when faced with challenging situations [10]. Individuals with high self-efficacy tend to have greater control over their environment and the challenging situations they encounter because they believe in their ability to influence the outcome of events and take initiative to make things happen [11]. They also tend to be more motivated by achieving their goals because they believe in their ability to succeed [12].

Nursing self-efficacy is an essential aspect of nursing practice that can influence patient outcomes [13,14,15], job satisfaction, stress levels, and overall performance [16,17]. Nurses with higher self-efficacy tend to have better coping mechanisms, take leadership roles [6,7], and provide patient-centred care [8]. To promote self-efficacy in nursing, nurses can engage in continuing education, set achievable goals, seek feedback and support from colleagues and supervisors [9], and work within a positive work culture [18].

For the assessment of nursing self-efficacy, self-report scales generally target specific tasks of clinical, managerial, or academic roles [13,14,15]. Although having specific scales is vital to exploring the specific relationships between task-specific self-efficacy and related outcomes, having a broader measure of nursing self-efficacy is also essential when establishing a relationship with broader outcomes (e.g., stress, burnout, and job dissatisfaction) and requires further research [19]. The most widely used self-report scale is the Nursing Profession Self-Efficacy Scale (NPSES), developed in 2016 and translated into several languages (e.g., Korean, Albanian, Turkish) [20,21,22].

The Nursing Profession Self-Efficacy Scale (NPSES) was initially developed in Italy by Caruso [13], and this scale is based on Bandura’s social cognition theory. The NPSES measures nurses’ professional activity, which includes evidence-based scientific knowledge and skills, ethical values and relationships, and collaboration with peers to meet patient needs [13].

The Albanian version of the scale of self-efficacy of the nursing profession (A-NPSES) from Duka et al. [20] is composed of 18 items, grouped into four subscales:

1. Factor 1 Nursing Care Procedure Situation (6 items) mainly addresses self-efficacy related to nurses’ skills in various clinical situations.
2. Factor 2 Nursing Research Situation (3 items) mainly addresses self-efficacy associated with research-based nursing practices.
3. Factor 3 Nursing Ethics Situation (5 items) addresses self-efficacy related to nursing ethical issues.
4. Factor 4 Nursing Practice Situation (4 items) addresses self-efficacy related to different nursing practices in different clinical contexts.

Each item has a response mode with a five-step Likert scale, ranging from one (not at all capable) to a maximum of five (fully capable). The calculation of the factors in the scale is as follows: Factor 1 = ((sum Items 1, 2, 3, 4, 5, and 6) − 6) × (100/30); Factor 2 = ((sum of elements 7, 8, and 9) − 3) × (100/15); Factor 3 = ((sum of elements 10, 11, 12, 13 and 14) − 5) × (100/25); and Factor 4 = ((sum of items 15, 16, 17 and 18) − 4) × (100/20), up to total score = ((sum of all items) − 18) × (100/90).

Self-efficacy (SE) has been identified as a factor that can influence nursing activities and nursing performance [23,24]. SE is, therefore, a fundamental aspect of the nursing profession, as it is closely related to decisions made in clinical nursing care [25]. SE is essential for nursing activities because it reflects the ability of nurses to verify nursing practices in different clinical contexts and affects the performance of nurses [26,27,28].

Nursing competence is a valuable resource for promoting equal access to quality care worldwide [29], so competence is essential to ensuring high-quality and safe nursing practice [30]. Furthermore, the nursing profession is multifaceted, requiring complex combinations of knowledge, skills, and attitudes, which enable a nurse to function effectively in a highly demanding environment.

To meet the demands of the changing healthcare environment, as well as those of patients, a competency-based approach has emerged as a critical policy in the nursing profession. This competency-based approach prepares nurses to face the complexity of their profession [31].

This study uses a new assessment tool, the Albanian version of the Nurse Professional Competence Scale Short Form (A-NPCS-SF), to measure self-reported professional competence among registered nurses. The psychometric properties of the NPCS-SF were comprehensively tested. The results showed that the NPCS-SF could contribute to safe and high-quality patient care by assessing nurses’ competencies from different perspectives [32].

The Albanian short version of the NPCS-SF scale was translated into Albanian in line with the recommendations by Beaton et al. [33] from Duka et al. [34].

The A-NPCS-SF is divided into six dimensions.

1. The first dimension, Nursing Care (Dimension 1), evaluates the quality of training procedures and how they can contribute to professional improvement.
2. The second dimension, Value-Based (Dimension 2), assesses nurses’ perceptions of the impact of professional ethics on responsible behaviour and ethical professional practice.
3. The third dimension, Medical Technical Care (Dimension 3), evaluates nurses’ perception of the acts put into their care practice from a medical and technical perspective.
4. The fourth dimension, Care Pedagogics (Dimension 4), evaluates the pedagogical contribution nurses must develop and have in clinical practice.
5. The fifth dimension, Documentation Administration (Dimension 5), evaluates nurses’ perceptions concerning the management of nursing documentation.
6. The sixth dimension, Leadership (Dimension 6), assesses nurses’ perception of good leadership development in coordinating care.

Each dimension has a score that is calculated using a formula application and the results of each dimension for the areas of expertise. The scale measures the above-mentioned four areas of expertise on a seven-point Likert scale (to a very low degree = one, to a low degree = two, to a relatively low degree = three, to neither a high nor low degree = four, to a relatively high degree = five, to a high degree = six, and to a very high degree = seven).

We needed to certify a specific scale in the nursing field to deal with their various demanding daily work challenges. Professional competencies and self-efficacy for nurses include sensitive topics for registered nurses [13]. Purposely, the role of a nurse includes professional nursing skills as an essential skill to ensure the best performance to help patients. Consequently, it is crucial to establish a solid education for registered nurses to define nursing competencies [35]. Continuous competency self-assessment allows nurses to reflect on their competencies and significantly impacts the quality of nursing practice and patient safety [36]. However, nursing competence is not necessarily a skill or task to be performed but rather a characteristic of adequate action in a specific nursing setting, defined by standard competencies [37]. In general terms, competence means feeling confident in the professional role in which nurses are involved in critical care. Most important is their ability to implement decision making quickly and efficiently in the face of life-threatening illnesses and to provide safe, high-quality care at all times [37]. Nurses have an essential role in promoting patients’ health, and it is paramount that they can perform their work optimally.

Our study had several objectives:

- Assess the level of professional competence of registered nurses in clinical practice through the Albanian version of the A-NPCS-SF;
- Evaluate the level of self-efficacy through the Albanian version of the A-NPSES;
- Evaluate whether there is a correlation between the development of professional competencies and the self-efficacy of Albanian nurses.

## 2. Materials and Methods

### 2.1. Study Design and Data Collection Procedures

We conducted a cross-sectional survey in several Albanian hospital settings based on a convenience sample of approximately 1154 registered nurses working in public and private hospitals in the 12 regions of Albania. A total of 3000 questionnaires were sent to participating hospitals between 25 January and 5 March 2022.

All study participants were nursing staff members with more than six months of work experience; all were directly involved in patient care. Participating hospitals were presented with two questionnaires, a consent form, and two self-addressed response envelopes.

Data were collected using anonymous, self-administered, and structured questionnaires. Completed questionnaires and consent forms were sealed and returned in self-addressed response envelopes. All completed questionnaires were protected confidentially, with no identifiable tags or specific personal information.

### 2.2. Statistical Analysis

SPSS statistical software for Windows, version 24 (SPSS Inc., Chicago, IL, USA) was used to analyse the data.

Following the user manual for the A-NPC-SF, responses to each skill area were recalculated to a score between 1 and 100, with 100 being the highest skill and 1 being the lowest. The same is true for the A-NPSES scale; responses to each area of self-efficacy were recalculated on a score ranging from 1 to 100, where 100 represents the highest self-efficacy, and 1 is the lowest.

Descriptive statistics were calculated, including means and standard deviations (SD), frequency, and percentages. Inferential statistics via one-way analysis of variance (ANOVA) were used to analyse the between-group means. Pearson’s correlation coefficient was calculated for the relationships between the A-NPC-SF factor scores to assess whether the tested sample exhibited the appropriate job skills based on their process. Pearson’s correlation was used to correlate continuous data.

A *p*-value less than 0.05 was considered statistically significant. Internal consistency of each skill area and full scale were calculated using Cronbach’s alpha. The missing values were replaced with the obtained mean of the missing elements. Cases with missing values for more than 50% of the responses were excluded from the study. The analyses were conducted independently by three authors.

### 2.3. Ethical Consideration

This study did not involve patients; the sample consisted of registered nurses. The study was designed, conducted, recorded, and reported consistently with international standards of scientific and ethical quality as indicated by Good Clinical Practice (GCP) and Standard Operating Procedures (SOP). Participants were also informed of the confidentiality and anonymity of their responses during the data collection and analysis processes. Ethical approval for this study was provided by the Ethics Committee of the Order of Albanian Nurses (0213.2023).

## 3. Results

In total, 985 of the 1154 participants completed the questionnaire (a response rate of 85.35%). The ages of the participants ranged from 21 to 62 years (the mean was 37.28 years ± 10.219 SD). The majority were women (82.9%). The detailed demographics are presented in Table 1.

The Cronbach’s alpha value for the A-NPCS-SF factors ranged from 0.932 to 0.953 and was 0.947 for the total scale. The Cronbach’s alpha value for the four A-NPSES factors ranged from 0.815 to 0.853 and was 0.875 for the total scale (Table 2).

Table 3 shows the correlations between the two scales used for our sample. Some of the scale sizes have significant correlations except the Nursing Ethics Situation dimension of the A-NPSES, which has no significant correlation with the other dimensions of the A-NPCS-SF.

The self-assessed competence scores of registered nurses participating in our study are described in Table 4. Table 4 compares the study participants’ professional competencies and self-efficacy according to their different characteristics.

The registered nurses had a mean total score for their professional skills of (M 83.93 ± 11.21805 SD) and a mean self-efficacy score of (M 87.85 ± 9.93427 SD). The highest score was obtained for the nurses with the most years of work in their registered operating unit (in the A-ANPCS-SF, it was M 91.92 ± 11.13 SD, while in the A-NPSES, it was M 98.25 ± 0.12 SD).

Specifically, regarding the size of the registered nurses’ professional skills, the highest average score was obtained for Years Profession vs. Medical Technical Care (M = 84.96 ± 12.75 SD; F = 2.578; *p* = 0.052), while the lowest average score was obtained for Job Satisfaction vs. Leadership (M = 81.70 ± 14.17 SD; F = 6.553; *p* = 0.000) (Table 5). Regarding the size of the self-efficacy scale, the nurses had significant scores only in Workplace (Ins Public) vs. Nursing Research Situation (M = 88.16 ± 12.17 SD; F = 9.499; *p* = 0.002) (Table 6).

## 4. Discussion

This study aimed to analyse the professional skills and self-efficacy levels of registered nurses enrolled in our investigation using the A-NPCS-SF and A-NPSES. The data collected showed an excellent average level of professional skills (with a range from 82.59 to 84.93) and self-efficacy (with a range from 88.03 to 90.31). This is in line with similar studies [38,39,40].

This could indicate a positive relationship between academic learning environments, in which professional competence and self-efficacy are learned and applied, and clinical learning environments, in which there is some intrinsic and/or extrinsic factor that increases the acquisition of these nursing skills [41,42].

From the results of our study, it is clear that the professional skills and self-efficacy of the analysed sample of registered nurses seem to follow the evolution of skills development. This is supported by statistically significant correlations between the partial scores of the dimensions in both the A-NPCS-SF and A-NPSES and between the different variables analysed (Table 4). In Table 4, we notice that Job Satisfaction is one of the variables analysed that has a positive statistical significance in the development of professional skills; this is more evident in Table 5, in which it is statistically significant in all dimensions of the A-NPCS-SF.

In fact, Job Satisfaction is a variable that has also been highlighted in other studies and can increase both effective nursing leadership and nursing care; in fact, in our investigation, the nurses obtained good scores (Job Satisfaction vs. Leadership: M = 81.7 ± 14.17 DS, F = 6.553, *p* = 0.000; and Job Satisfaction vs. Nursing Care: M = 83.30 ± 10.87 SD, F = 7.467, *p* = 0.000) [43,44].

Moreover, regarding Value-Based, this measure had a positive effect on Job Satisfaction, in line with other studies on this topic. This is fundamental because an efficient clinical practice based on these values requires nurses with more solid professional values and higher levels of professional satisfaction [45]. In our study, it has a statistically significant correlation with Job Satisfaction (M = 82.27 ± 11.83 SD; F = 7.607; *p* = 0.000).

The other dimensions, Medical Technical Care, Care Pedagogics, and Documentation Administration of the A-NPCS-SF scale, also had a statistically significant correlation with Job Satisfaction with a range of *p* from 0.001 to 0.002.

Meanwhile, for the A-NPSES, we had a statistically positive correlation between Workplace and Nursing Research Situation (M = 88.16 ± 12.17 SD; F = 9.499; *p* = 0.002). This is important because nursing research improves workplace self-efficacy [46,47,48].

Another aim of our study was to assess whether there was a correlation between professional skills development and self-efficacy. In our analysis, except for the Nursing Ethics Situation dimension of the A-NPSES, all other dimensions of the A-NPSES correlated positively with the A-NPCS-SF. We can deduce that self-efficacy positively influences the development of professional skills and, therefore, affects whether a nurse professionally trained and prepared for different contexts of clinical practice, which is in agreement with other studies [49,50,51].

Our research shows that more than one skill dimension on the A-NPCS-SF scale positively correlated with Job Satisfaction. This fulfilled the authors’ goal of having the broadest possible overview of the topic. There are different constituents of professional skills and self-efficacy because people are naturally different in terms of their traits, characteristics, and communication skills. It is, therefore, necessary for a nurse to understand when a particular ability, skill, attitude, or knowledge of fundamental characteristics of competencies must be demonstrated in clinical practice [52]. This is also based on every nurse’s self-efficacy, which is the perception of their ability to carry out their nursing activities professionally.

In addition, our findings reveal that we still need to analyse what other determinants may affect nursing skills. This reflects the need to produce further research that, by investigating a broader spectrum of people, would allow us to shed light on a theme still largely empirically founded and with little scientific evidence. The fact that our study has led to positive results in terms of job satisfaction proves that different cultures, contexts, and individuals require different behaviours and methods that vary over time, depending on the situation.

Job satisfaction is a key factor in creating a positive relationship for patient safety through a positive cultural attitude also due to the possession of professional competences. Nursing training should focus not only on providing appropriate professional competences, but also on culture and strengthening nurses’ job satisfaction to help them stay positive throughout their career. A culture of positive job satisfaction among employees is also important when new nurses join an organization.

We strongly advise both educators and nursing managers to educate nurses from a professional point of view, but also to provide adequate training to help them recognize and improve their perception of job satisfaction, since a good organizational climate generates greater satisfaction. Through positive job satisfaction, nurses can develop better standards and operational processes, enabling patients to receive the best-quality care. Ultimately, job satisfaction influences nurses’ attitudes towards patient safety and the quality of care provided. Our findings warrant future studies on the positive effect of job satisfaction on nurses.

### Study Limitation and Strenghts

Our study has the inherent limitations of a correlation study. Its cross-sectional design makes determining the causal relationships between study variables impossible. Care should be taken in assuming a causality between an attitude of nursing competence and patient safety and its predictors. Furthermore, given social desirability, biases, and common variances, self-reported measures using self-administered questionnaires may have influenced the data results. Furthermore, the sample analysed was of convenience. Therefore, we cannot generalize the data; future research should be aimed at improving these aspects. In particular, the external generalizability of the current findings may be limited because the questionnaires were collected from hospital nurses. However, these findings warrant further investigations of job satisfaction in other types of nursing professions.

The strength of our study is that it is the first investigation in Albania to correlate professional skills and self-efficacy. In addition, our study highlights the statistically significant correlation of job satisfaction with professional competencies; future investigations should be directed to identify possible predictors.

## 5. Conclusions

The results of this analysis showed that there is only a one-dimensional correlation between self-efficacy and professional competence, particularly between Workplace (a-NPSES) and Nursing Research Situation (A-NPCS-SF); this shows that self-efficacy has little effect in the development of professional competencies.

This is probably because, according to some authors, self-efficacy has to do with one’s self-perception of competence rather than their actual level of competence. However, the results of our investigation have highlighted the importance of a close association between job satisfaction and professional skills development.

## Figures and Tables

**Table 1:** Socio-demographic data (*n* = 985).

Gender		
	*n*	%
F	817	82.9
M	168	17.1
**Workplace**		
Private Health Institution	12	1.2
Public Health Institution	973	98.8
**Degree**		
Private University	208	21.1
Public University	777	78.9
**Age Classes**		
20–30	316	32.1
31–41	333	33.8
41–52	195	19.8
53–63	103	10.5
NA	38	3.9
**Years in Profession**		
0–11	546	55.4
12–23	260	26.4
24–35	120	12.2
36–47	37	3.8
NA	22	2.2
**Years in Ward Unit**		
0–13	734	74.5
14–27	149	15.1
28–41	42	4.3
42–55	2	0.2
NA	58	5.9
**Post-Graduate Education**		
Master 1	776	78.8
Master 2	141	14.3
Other Degree	11	1.1
NA	57	5.8
**Job Satisfaction**		
Nothing	16	1.6
Little	74	7.5
Fair	410	41.6
More	468	47.5
NA	17	1.7

**Table 2:** The mean and Cronbach’s alpha of the factors between the Albanian version of Nurse Professional Competence Scale Short Form (A-NPCS-SF) and the Albanian version of Nursing Profession Self-Efficacy Scale (A-NPSES) (*n* = 895).

Scale	Dimension	Mean	SD	Alpha di Cronbach	Total Alpha di Cronbach
**A-NPCS-SF**	1—Nursing Care	84.93	11.30	0.933	0.947
	2—Value-Based	83.83	12.06	0.935	
	3—Medical Technical Care	82.59	13.56	0.937	
	4—Care Pedagogics	84.28	12.49	0.933	
	5—Documentation Administration	84.53	12.01	0.932	
	6—Leadership	83.40	14.08	0.953	
**A-NPSES**	1—Nursing Care Procedure Situation	90.31	10.35	0.828	0.875
	2—Nursing Research Situation	88.03	12.28	0.853	
	3—Nursing Ethics Situation	88.08	10.95	0.815	
	4—Nursing Practice Situation	84.97	12.84	0.868	

**Table 3:** The correlations between the A-NPCS-SF and the A-NPSES (*n* = 895).

		ANPC Mean	Nursing Care	Value-Based	Medical Technical Care	Care Pedagogics	Documentation Administration	Leadership	ANPSES Mean	Nursing Care Procedure Situation	Nursing Research Situation	Nursing Ethics Situation	Nursing Practice Situation
ANPC Mean	r	1											
	*p*												
Nursing Care	r	0.920 **	1										
	*p*	0.000											
Value-Based	r	0.899 **	0.804 **	1									
	*p*	0.000	0.000										
Medical Technical Care	r	0.895 **	0.779 **	0.835 **	1								
	*p*	0.000	0.000	0.000									
Care Pedagogics	r	0.915 **	0.837 **	0.787 **	0.790 **	1							
	*p*	0.000	0.000	0.000	0.000								
Documentation Administration	r	0.922 **	0.818 **	0.793 **	0.796 **	0.835 **	1						
	*p*	0.000	0.000	0.000	0.000	0.000							
Leadership	r	0.813 **	0.719 **	0.619 **	0.599 **	0.669 **	0.713 **	1					
	*p*	0.000	0.000	0.000	0.000	0.000	0.000						
ANPSES Mean	r	0.055	0.031	0.070 *	0.063 *	0.061	0.041	0.027	1				
	*p*	0.085	0.326	**0.027**	**0.047**	0.057	0.202	0.393					
Nursing Care Procedure Situation	r	0.067 *	0.043	0.071 *	0.076 *	0.074 *	0.053	0.041	0.868 **	1			
	*p*	**0.035**	0.176	**0.026**	**0.017**	**0.020**	0.097	0.202	0.000				
Nursing Research Situation	r	0.048	0.029	0.070 *	0.058	0.046	0.043	0.014	0.843 **	0.642 **	1		
	*p*	0.130	0.367	**0.027**	0.067	0.152	0.181	0.650	0.000	0.000			
**Nursing Ethics Situation**	r	0.010	−0.007	0.027	0.023	0.025	0.005	−0.017	0.889 **	0.776 **	0.667 **	1	
	*p*	0.748	0.826	0.393	0.478	0.426	0.883	0.594	0.000	0.000	0.000		
Nursing Practice Situation	r	0.061	0.041	0.070 *	0.060	0.063 *	0.038	0.052	0.831 **	0.606 **	0.566 **	0.637 **	1
	*p*	0.056	0.203	**0.028**	0.061	**0.049**	0.228	0.101	0.000	0.000	0.000	0.000	

**. The correlation is significant at a level of 0.01 (two-tailed). *. The correlation is significant at a level of 0.05 (two-tailed).

**Table 4:** Correlation between the characteristics of registered nurses and the A-NPCS-SF and the A-NPSES.

		A-NPCS-SF				A-NPSES			
		Mean ± SD		F	*p*	Mean ± SD		F	*p*
Age Classes	20–30	83.16	11.70	1.617	0.184	87.47	9.88	0.421	0.738
	31–41	84.17	11.19			87.80	9.70		
	41–52	83.28	11.23			88.27	10.08		
	53–63	85.75	10.28			88.50	10.30		
	Total	83.82	11.28			87.86	9.90		
Years in Profession	0–11	83.10	11.58	2.500	0.058	87.29	9.87	1.214	0.303
	12–23	84.71	10.62			88.61	9.77		
	24–35	85.22	11.10			87.95	11.00		
	36–47	86.32	10.09			88.90	7.87		
	Total	83.92	11.24			87.79	9.93		
Years in Ward Unit	0–13	84.02	11.28	0.506	0.678	87.77	9.76	1.254	0.289
	14–27	83.38	11.56			88.58	9.96		
	28–41	84.54	11.13			86.55	13.06		
	42–55	91.92	2.83			98.25	0.12		
	Total	83.96	11.30			87.86	9.96		
Gender	F	84.04	10.87	0.483	0.487	87.85	9.96	0.000	0.997
	M	83.38	12.80			87.84	9.82		
	Total	83.93	11.22			87.85	9.93		
Workplace	Ins Private	77.54	14.40	3.952	0.047	83.47	9.26	2.367	0.124
	Ins Public	84.00	11.16			87.90	9.93		
	Total	83.93	11.22			87.85	9.93		
Job Satisfaction	Nothing	91.75	5.86	7.493	0.000	90.94	9.08	0.760	0.517
	Little	85.91	10.76			88.02	9.55		
	Fair	82.45	11.05			88.01	9.81		
	More	85.02	10.97			87.48	10.18		
	Total	84.11	11.04			87.80	9.96		

**Table 5:** Correlation between the characteristics of registered nurses and the dimensions of the A-NPCS-SF and the A-NPSES.

		A-NPCS-SF																							
		Nursing Care				Value-Based				Medical Technical Care				Care Pedagogics				Documentation Administration				Leadership			
		Mean ± SD		F	*p*	Mean ± SD		F	*p*	Mean ± SD		F	*p*	Mean ± SD		F	*p*	Mean ± SD		F	*p*	Mean ± SD		F	*p*
Age Classes	20–30	84.13	11.56	1.543	0.202	83.63	12.73	1.547	0.201	81.58	14.25	1.111	0.344	83.33	13.11	1.305	0.272	84.00	12.62	1.885	0.130	82.28	15.13	1.394	0.243
	31–41	85.21	11.60			84.13	11.78			82.87	13.35			84.44	12.64			84.74	11.64			83.61	13.53		
	41–52	84.37	11.15			82.43	12.29			82.29	13.46			83.88	12.41			83.42	12.08			83.32	14.67		
	53–63	86.68	10.35			85.41	10.52			84.20	12.72			86.04	10.91			86.70	11.66			85.45	11.66		
	Total	84.84	11.37			83.75	12.09			82.47	13.61			84.13	12.58			84.43	12.08			83.31	14.15		
Years Profession	0–11	84.21	11.62	1.892	0.129	83.37	12.53	1.229	0.298	81.59	14.10	2.578	**0.052**	83.34	12.85	2.128	0.095	83.72	12.25	2.132	0.095	82.38	14.66	3.089	**0.026**
	12–23	85.81	10.87			83.83	11.63			83.35	12.61			85.10	11.82			85.31	11.51			84.87	13.18		
	24–35	85.89	10.96			85.26	11.48			**84.96**	**12.75**			85.37	12.78			85.30	11.80			**84.53**	**13.41**		
	36–47	86.84	10.55			86.01	9.72			83.69	13.19			86.67	10.93			87.55	12.33			87.20	10.66		
	Total	84.95	11.32			83.83	12.07			82.56	13.55			84.20	12.53			84.50	12.02			83.50	14.03		
Years Ward Unit	0–13	84.95	11.44	0.454	0.715	84.24	12.14	0.741	0.528	82.76	13.47	0.232	0.874	84.27	12.62	0.511	0.675	84.64	11.97	0.662	0.576	83.29	14.35	0.733	0.532
	14–27	84.39	11.41			82.82	12.06			81.83	14.47			83.61	12.83			83.90	12.55			83.73	13.83		
	28–41	85.83	11.19			83.95	11.49			82.26	14.34			85.26	11.32			85.18	12.25			84.78	12.22		
	42–55	91.96	8.84			90.18	13.90			85.71	0.00			92.58	1.84			94.64	2.53			96.43	5.05		
	Total	84.92	11.41			84.01	12.09			82.59	13.65			84.23	12.58			84.56	12.07			83.46	14.17		
Gender	F	85.01	10.94	0.255	0.613	83.86	11.90	0.042	0.838	82.57	13.40	**0.007**	0.933	84.53	11.90	1.912	0.167	84.66	11.65	0.584	0.445	83.59	13.80	0.895	0.344
	M	84.53	12.94			83.66	12.88			**82.67**	**14.32**			83.06	15.01			83.89	13.62			82.46	15.32		
	Total	84.93	11.30			83.83	12.07			82.59	13.55			84.28	12.49			84.53	12.01			83.40	14.07		
Workplace	Ins Private	80.17	13.11	2.158	0.142	76.15	16.27	4.940	**0.026**	75.57	15.89	3.264	0.071	74.82	17.45	7.016	**0.008**	78.84	15.39	2.734	0.099	79.67	11.68	0.854	0.356
	Ins Public	84.99	11.28			**83.92**	**11.99**			82.67	13.51			**84.39**	**12.38**			84.60	11.95			83.44	14.10		
	Total	84.93	11.30			83.83	12.07			82.59	13.55			84.28	12.49			84.53	12.01			83.40	14.07		
Job Satisfaction	Nothing	91.15	8.09	7.467	**0.000**	93.08	8.35	7.607	**0.000**	90.01	6.52	5.803	**0.001**	92.98	5.87	4.880	**0.002**	89.51	7.77	5.101	**0.002**	93.75	6.84	6.553	**0.000**
	Little	86.66	11.57			85.60	12.62			85.44	10.81			86.96	10.03			86.71	11.73			84.09	17.83		
	Fair	**83.30**	**10.87**			**82.27**	**11.83**			**81.01**	**13.50**			**83.33**	**11.62**			**83.09**	**12.06**			**81.70**	**14.17**		
	More	86.25	11.12			84.97	11.72			83.71	13.46			84.78	13.21			85.63	11.71			84.78	13.08		
	Total	85.12	11.12			84.01	11.91			82.80	13.31			84.47	12.32			84.70	11.89			83.57	14.01		

**Table 6:** Correlation between the characteristics of registered nurses and the dimensions of the A-NPSES.

		A-NPSES Scale															
		Nursing Care Procedure Situation				Nursing Research Situation				Nursing Ethics Situation				Nursing Practice Situation			
		Mean ± SD		F	*p*	Mean ± SD		F	*p*	Mean ± SD		F	*p*	Mean ± SD		F	*p*
Age Classes	20–30	89.74	10.52	0.833	0.476	87.70	12.11	0.294	0.830	87.77	10.85	0.471	0.703	84.68	12.61	0.445	0.721
	31–41	90.45	10.17			87.99	12.17			87.90	10.80			84.85	12.78		
	41–52	90.48	10.24			88.14	13.00			88.57	10.92			85.90	13.14		
	53–63	91.52	10.40			89.00	11.83			88.97	11.55			84.51	12.02		
	Total	90.33	10.33			88.03	12.28			88.11	10.92			84.97	12.82		
Years Profession	0–11	89.73	10.63	1.171	0.320	87.41	12.26	1.355	0.255	87.50	10.85	1.618	0.183	84.52	12.03	1.006	0.389
	12–23	91.06	9.56			88.23	12.64			89.09	10.65			86.06	13.08		
	24–35	90.58	11.28			88.83	12.53			87.53	12.35			84.83	13.20		
	36–47	91.35	8.52			90.99	9.06			89.73	9.50			83.51	11.36		
	Total	90.26	10.37			87.95	12.30			88.02	10.96			84.94	12.82		
Years Ward Unit	0–13	90.19	10.37	0.399	0.754	87.94	12.17	1.086	0.354	87.96	10.75	1.089	0.353	84.98	12.66	1.327	0.264
	14–27	90.58	10.13			89.08	11.78			88.99	11.11			85.67	12.81		
	28–41	88.97	13.50			87.14	14.15			86.86	14.12			83.21	14.64		
	42–55	95.00	2.36			100.00	0.00			98.00	2.83			100.00	0.00		
	Total	90.20	10.48			88.11	12.20			88.10	10.98			85.04	12.78		
Gender	F	90.37	10.26	0.143	0.705	88.06	12.39	0.039	0.844	87.95	11.02	0.635	0.426	85.01	12.84	0.039	0.844
	M	90.04	10.77			87.86	11.70			88.69	10.63			84.79	12.88		
	Total	90.31	10.35			88.03	12.27			88.08	10.95			84.97	12.84		
Workplace	Ins Private	89.72	8.10	0.040	0.842	77.22	15.94	9.499	0.002	87.33	9.47	0.056	0.813	79.58	14.05	2.141	0.144
	Ins Public	90.32	10.38			88.16	12.17			88.09	10.97			85.04	12.82		
	Total	90.31	10.35			88.03	12.27			88.08	10.95			84.97	12.84		
Job Satisfaction	Nothing	93.54	9.31	0.947	0.417	92.92	8.60	1.030	0.378	90.75	10.90	0.540	0.655	86.56	10.91	0.747	0.524
	Little	90.81	9.39			87.03	13.51			88.49	10.26			85.74	11.55		
	Fair	90.52	9.99			87.82	12.53			88.22	11.08			85.48	12.71		
	More	89.84	10.90			88.03	12.00			87.72	10.97			84.35	13.23		
	Total	90.26	10.39			87.95	12.30			88.04	10.96			84.97	12.85		

## Data Availability

The data presented in this study are available within the article.
